# A Novel Cold-Adapted Lipase from *Sorangium cellulosum* Strain So0157-2: Gene Cloning, Expression, and Enzymatic Characterization

**DOI:** 10.3390/ijms12106765

**Published:** 2011-10-13

**Authors:** Yuan-Yuan Cheng, Yun-Kai Qian, Zhi-Feng Li, Zhi-Hong Wu, Hong Liu, Yue-Zhong Li

**Affiliations:** State Key Laboratory of Microbial Technology, School of Life Science, Shandong University, Jinan 250100, China; E-Mails: cyysdu@gmail.com (Y.-Y.C.); qyk-1@163.com (Y.-K.Q.); lizhifeng@sdu.edu.cn (Z.-F.L.); wuzhihong@sdu.edu.cn (Z.-H.W.); liuhong05@sdu.edu.cn (H.L.)

**Keywords:** *Sorangium cellulosum*, cold-adapted lipase, detergent tolerant, organic synthesis

## Abstract

Genome sequencing of cellulolytic myxobacterium *Sorangium cellulosum* reveals many open-reading frames (ORFs) encoding various degradation enzymes with low sequence similarity to those reported, but none of them has been characterized. In this paper, a predicted lipase gene (*lipA*) was cloned from *S. cellulosum* strain So0157-2 and characterized. *lipA* is 981-bp in size, encoding a polypeptide of 326 amino acids that contains the pentapeptide (GHSMG) and catalytic triad residues (Ser114, Asp250 and His284). Searching in the GenBank database shows that the LipA protein has only the 30% maximal identity to a human monoglyceride lipase. The novel *lipA* gene was expressed in *Escherichia coli* BL21 and the recombinant protein (r-LipA) was purified using Ni-NTA affinity chromatography. The enzyme hydrolyzed the *p*-nitrophenyl (*p*NP) esters of short or medium chain fatty acids (≤C_10_), and the maximal activity was on *p*NP acetate. The r- LipA is a cold-adapted lipase, with high enzymatic activity in a wide range of temperature and pH values. At 4 °C and 30 °C, the *K*_m_ values of r-LipA on *p*NP acetate are 0.037 ± 0.001 and 0.174 ± 0.006 mM, respectively. Higher pH and temperature conditions promoted hydrolytic activity toward the *p*NP esters with longer chain fatty acids. Remarkably, this lipase retained much of its activity in the presence of commercial detergents and organic solvents. The results suggest that the r-LipA protein has some new characteristics potentially promising for industrial applications and *S. cellulosum* is an intriguing resource for lipase screening.

## 1. Introduction

Lipases/esterases are members of the α/β hydrolase superfamily, which is characterized by having a semi-conserved pentapeptide domain GXSXG (X represents any amino acid) and the Ser, Asp (or Glu), His catalytic triad [1,2]. The enzymes catalyze the hydrolysis and synthesis of fatty acid esters [3,4]. Despite sharing a common catalytic mechanism and a similar structure, the amino acid sequences of different lipases/esterases are greatly variable [5], which endow them potential specific catalytic activities on different ester substrates or functions under various tolerable conditions. Although extensively present in different organisms, lipases/esterases from microorganisms are more commercially significant for their versatile application potential in laundry, food, oil chemistry, fine chemistry, pharmaceutical and paper industries, as well as in biodiesel production, waste treatment and other biotechnological applications [6–9]. Lipases that are adapted to cold temperatures or that are generally thermostable are of particular interest for industrial processes.

In the last two decades, a great number of cold-adapted lipase genes have been cloned and many related researches have been conducted. In the majority of cold-adapted lipases, the optimum catalytic activity was around 20–40 °C and are stable at a wide range of temperatures. However, these cold-adapted enzymes are unstable above 65 °C [1]. Cold-adapted lipases are attractive biocatalysts in biotechnology because they can be used at lower temperatures as additives in the food industry and in laundry detergents to allow washing in cold water [10–13]. Additionally, cold-adapted lipases have the potential to be used as catalysts in the organic synthesis of chiral intermediates, allowing relatively unstable compounds to be produced at low temperatures [14].

Myxobacteria are gram-negative gliding bacteria that have complicated social lives and excellent ability to produce various bioactive com pounds [15,16]. Among different myxobacterial species, the cellulolytic myxobacterium *Sorangium cellulosum* is extremely interesting in drug screening, and almost half of the discovered myxobacterial secondary metabolites are produced by different strains of this species [17]. In addition to these defined characteristics, *S. cellulosum* also has excellent and extensive degradation ability on various macromolecules, including many different kinds of polysaccharide and lipid, of which the involved enzymes have been less investigated. *S. cellulosum* has the known largest bacterial genome, reaching 13.1 Mb in the sequenced So ce56 strain [18]. Consistent with the extensive degradation ability on a wide range of macromolecules, the *S. cellulosum* genome contains many open-reading frames (ORFs) predicted encoding different hydrolytic enzymes. For instance, 45 ORFs on So ce56 genome were predicted to encode various lipases/esterases (EC 3.1.1.). The predicted products of these ORFs are all in low similarity to those studied lipases/esterases and none of them has been characterized, suggesting their potentially specific characteristics. In the present study, to explore characteristics and commercial significance of *S. cellulosum* degradation enzymes, for the first time, we cloned a lipase ORF from strain So0157-2 and expressed it in *Escherichia coli* BL21. Characterization of the recombinant enzyme suggests that the purified r-LipA is a cold-adapted lipase which has some new characteristics potentially promising for industrial applications and the myxobacterial species *S. cellulosum* is an intriguing resource for lipase screening.

## 2. Results and Discussion

### 2.1. Gene Cloning and Sequence Analysis

Using the primer pair of LipA-F (5′-GGAATTCCATATGATGCCCGCGGACACCTTCACGTTTC AG-3′) and LipA-R (5′-CCCAAGCTTGCCGGCCGCGGCGCCGGCGC-3′), a 981-bp open reading frame (ORF), designated *lipA*, was amplified from the genomic DNA of So0157-2. There is a putative Shine-Dalgarno sequence (SD), AGGG, ten bases upstream of the ATG start codon of the gene. The ORF encoded a predicted protein of 326 amino acids with a calculated molecular mass of 35.6 kDa and a theoretical isoelectric point of 8.58. In addition, no signal peptide was predicted by SignalP 3.0 [19]. The nucleotide sequence for the *S. cellulosum* So0157-2 lipase gene (*lipA*) has been deposited in the GenBank database under accession number JF739860.

**Figure 1:**
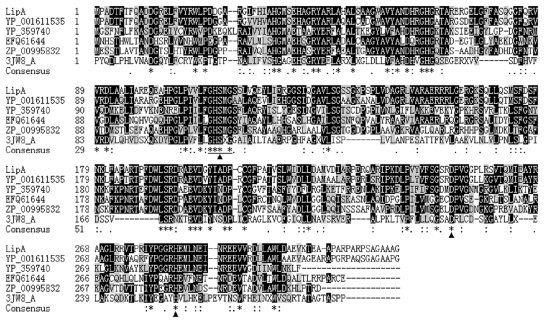
Multiple sequence alignment of LipA and other related proteins. Sequences alignment was carried out using ClustalW program [25]. The conserved Gly-X-Ser-X-Gly motif was underlined, and the serine, aspartic acid and histidine catalytic triad was pointed out by filled triangles obtained from 3JW8_A and other enzymes. LipA: from *S. cellulosum* So0157-2 (this study); YP_001611535, predicted lysophospholipase from *S. cellulosum* So ce56; EFQ61644, alpha/beta fold family hydrolase from *Pseudomonas fluorescens* WH6; ZP_00995832, Lysophospholipase L2 from *Janibacter* sp. HTCC2649; YP_359740, alpha/beta fold family hydrolase from *Carboxydothermus hydrogenoformans* Z-2901; 3JW8_A, chain A of crystal structure of monoglyceride lipase from human.

The deduced amino acid sequence of LipA is highly similar (84% identity) to its homologue in *S. cellulosum* So ce56 (GenBank: YP_001611535, predicted to be a hydrolase (EC 3.1.1.) with lysophospholipase function). Searching in the GenBank database shows that LipA has low similarity to those studied lipases with a maximum identity of 30% to a human monoglyceride lipase (3JW8_A) [20]. Multiple sequence alignment reveals that the LipA sequence contains the typical penta-peptide consensus residues GHSMG (at the position of 112–116 sites) and the catalytic triad (Ser114, Asp250 and His284) (Figure 1), which is essential for lipolytic activity [20,21].

### 2.2. Expression and Purification of the r-LipA

**Figure 2:**
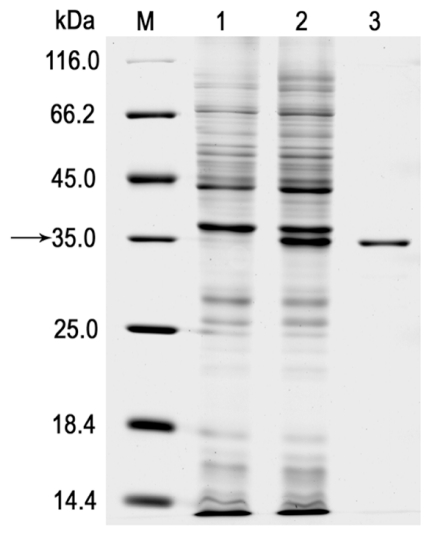
SDS-PAGE of the expression and purification of r-LipA in *E. coli*. Lane M: molecular weight protein marker; lane 1: total protein extract of induced culture of *E. coli*/pET-22b (+), as negative control; lane 2: total protein extract of induced culture of *E. coli*/pET-lipA; lane 3: purified r-LipA after Ni-NTA affinity chromatography.

The *lipA*-containing plasmid, pET-*lipA*, was expressed in *E. coli* BL21 (DE3). The recombinant LipA (r-LipA) was in a soluble form, which was further purified using Ni-NTA affinity chromatography [22,23]. SDS-PAGE analysis of the purified r-LipA showed a single band corresponding to approximate 35.6 kDa (Figure 2), which is consistent with the calculated molecular mass of the recombinant protein. The purified protein was further analyzed using LC-ESI-MS/MS. The GELGFFASQGGFQR and SVTQMIEAYR peptides matched the deduced amino acid sequence of LipA, indicating that the purified protein was the heterogeneously expressed r-LipA. The hydrolytic activity of the crude enzyme, extracted from broken cells, was 0.201 ± 0.003 U/mg at 30 °C on *p*NP acetate, whereas the specific activity of the purified r-LipA was 4.290 ± 0.075 U/mg. After dialysis and concentration, the overall enzyme yield was 64.41% (calculated from the activity ratio of the purified protein and the supernatant of the lysates), with a 20.91-fold enrichment. Esterases and lipases (EC 3.1.1.) share the semi-conserved pentapeptide domain GXSXG and the Ser, Asp (or Glu), His catalytic triad [1], and are both able to hydrolyze ester bonds. They can be distinguished by the degradation test of a triglyceride derivative 1,2-di-*O*-lauryl-rac-glycero-3-glutaric acid 6′-methylresorufin ester (DGGR) [6,24]. The DGGR degradation test showed that r-LipA had a similar hydrolytic activity against DGGR as the defined lipase from *Candida rugosa* (the positive control of the method), forming the chromogenic product, methylresorufin. The catalytic activities of r-LipA and the *Candida rugosa* lipase on DGGR were 15.49 ± 0.45 U/L and 8.92 ± 0.15 U/L after 4-h incubation at 30 °C, respectively; whereas the inactivated r-LipA protein did not hydrolyze DGGR. The result indicated that the r-LipA protein is truly a lipase enzyme.

### 2.3. Effects of Temperature on the r-LipA Avtivity and Stability

**Figure 3:**
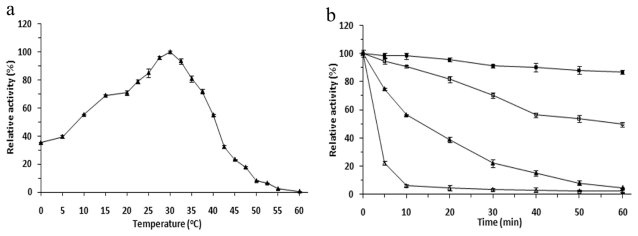
Effects of temperature on activity and stability of the r-LipA. (**a**) Effect of temperature on r-LipA activity. Enzymatic activity of purified lipase (0.5 mg/mL) was determined in a temperature range of 20–60 °C in 50 mM Tris-HCl (pH 8.0); (**b**) Thermostability of r-LipA. The enzyme was pre-incubated at 40 °C (filled square), 50 °C (empty square), 55 °C (filled triangle), or 60 °C (empty triangle) in 50 mM Tris-HCl (pH 8.0), and aliquots were withdrawn at regular intervals to measure residual activity at 30 °C. All measurements were carried out in triplicate.

Effects of temperatures on enzymatic activity of r-LipA were measured in a temperature range between 0 °C and 60 °C with *p*NP acetate as the substrate. The protein exhibited the maximum activity at 30 °C and the relative activity still reached to 35.2% even at 0 °C. The activity rapidly decreased at temperatures greater than 45 °C and was almost completely lost at 60 °C (Figure 3a). The r-LipA was, respectively, 86.62% and 49.58% active after 1 h of incubation at 40 °C and 50 °C. However, it lost nearly 80% of activity after 30-min pre-incubation at 55 °C and the half-life was just 10 min (Figure 3b). According to the presently prevalent definition [26], we define our protein a cold-adapted enzyme. Among the studied cold-adapted lipases known so far, LipA from *Acinetobacter* sp. XMZ-26 had the lowest optimum temperature (15 °C) for its enzymatic activity [13], while the most of cold-adapted lipases showed the the hightest temperature optimum (20–40 °C) [1]. The optimum temperature for the r-LipA was 30 °C. Similar to some of the studied cold-adapted lipases, r-LipA had no detectable lipase activity at 60 °C and is unstable above 60 °C. The LipA1 from *Psychrobacter* sp.7195 exhibited 18% and 35% activity at 0 °C and 10 °C, respectively [27], EML1 from a deep-sea sediment metagenome exhibited 60% of the maximal activity at 5 °C [28], and rLipA from *Pseudomonas* sp. 7323 showed 15% and 30% activity at 0 °C and 10 °C, respectively [29]. Similar to these cold-adapted lipase, the r-LipA from So0157-2 retained 35.2% and 55.2% of the peak activity at 0 °C and 10 °C, respectively. These results indicate that r-LipA is a cold-adapted lipase. It is known that myxobacterial strains normally apt to live in <30 °C environments [30]. Cold-active enzymes were probably for their ecological adaptative survivals.

### 2.4. Effects of pH on the r-LipA Avtivity and Stability

**Figure 4:**
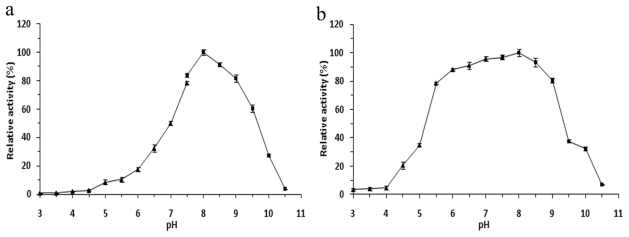
Effect of pH on activity and stability of r-LipA. (**a**) Effect of pH on r-LipA activity. The reaction was carried out at 30 °C in buffers with pH ranging from 3.0 to 10.5; (**b**) pH stability of r-LipA. The residual activity of r-LipA was determined at 30 °C after 8 h pre-incubation at 4 °C in buffers ranging from pH 3.0 to 10.5. The following buffers were used: McIlvaine buffer (pH 3.0–7.5, filled triangle); 50 mM Tris-HCl buffer (pH 7.5–10.5, filled square). All measurements were carried out in triplicate.

In contrast to the cold-adaptation characteristics, the lipase retained high activity in a broad pH range (3.0–10.5), and reached the maximum at pH 8.0 (Figure 4a). 8-h incubation in buffers between pH 6.0 and pH 9.0 decreased less than 20% of the enzymatic activity (Figure 4b). These data suggested that r-LipA showed the optimum activity and stability in alkalescent pH conditions.

### 2.5. Effects of Metal Ions, Detergents and Organic Solvents on the r-LipA Activity

The activity of r-LipA in the presence of different metal ions, detergents and organic solvents are shown in Tables 1, 2 and 3, respectively. The enzyme activity was strongly inhibited by Cu^2+^ and Zn^2+^ even at the lowest tested concentration (1 mM) and little or not affected by Li^+^, Na^+^, K^+^, Co^2+^ and ethylenediamine tetraacetic acid (EDTA). The enzyme activity was enhanced by the presence of Ca^2+^ and Mg^2+^ at a concentration of 10 mM, approximately1.4-, 1.26-fold, respectively. Interestingly, Cr^3+^ and Fe^2+^ increased the activity by about 10% and 40% at a concentration of 1 mM but strongly inhibited the activity at a concentration of 10 mM. The enzymatic activity of r-LipA was strongly inhibited by Ni^2+^, Hg^2+^ only at high concentration (10 mM).

The r-LipA protein exhibited wide tolerances against various conditions, which are promising for industrial applications. Lipases and esterases are commonly used as additives in detergents, mainly in household and industrial laundry and in household dishwashers [31]. The r-LipA was found to be able to retain a high level of activity in the presence of many commercially available detergents. In the tested detergents, the addition of other tested non-ionic detergents (Tween20, Tween80, Triton X-100) had positive effects on the r-LipA activity to different extents. For example, 0.1% concentration of Triton X-100 produced approximate 150% enzymatic activity on *p*NP acetate, and 1% concentration gave an increase of the activity to approximately 200%. In contrast, the ionic detergent SDS sharply decreased the hydrolytic activity of r-LipA to approximately 10%, and changing SDS concentration from 0.1% to 1% changed its effects marginally. However, low concentration (0.1%) of ionic detergent CTAB gave approximate 200% activity on *p*NP acetate, compared to the control; whereas increasing the concentration to 1% had almost no effects on the enzymatic activity. These results indicate that this cold-adapted lipolytic enzyme may be useful in detergents designed to be used at cold temperatures.

Activity in organic solvents is an important property of protein catalysts used in organic synthesis reactions. To assess the potential of r-LipA this context, its activity in selected sixteen solvents of different logarithm of partition coefficient (log *P*) values was examined. The log *P* value is defined as the logarithm of its partition coefficient in standard *n*-octane/water two phase systems [31,32]. In the test, the presence of many organic solvents increased the hydrolytic activity. For example, *n*-Heptane (log *P* = 4.0) and isooctane (log *P* = 4.7) enhanced the lipase activity of r-LipA to approximately 1.6-fold after 30 min incubation. Because polar solvents are water-miscible, and are able to strip off the essential water layer of the protein [33], the organic solvents with bigger logarithms of partition coefficient (log *P*s) are more hydrophobic, and thus are better for non-aqueous enzymology [34]. Therefore, organic solvents with log *P* values <2.0 are generally not considered for biocatalysis [35]. However, although dimethyl sulfoxide and methanol have low log *P*s (<2.0), r-LipA is very stable in the presence of these two organic solvents. Changing the incubation time from 30 min to 2 h had small changes of the effects of solvent on enzymatic activity. That r-LipA demonstrated increased activity following exposure to such solvents suggests that it might be a useful catalyst in organic solvent systems.

### 2.6. Substrate Specificity of the r-LipA

The substrate specificity of r-LipA against *p*NP fatty acyl esters with various lengths of the acyl chain was assayed at pH 8.0 and 30 °C (Table 4). The r-LipA exhibited high activity against short-chain fatty acids, and the activity significantly decreased with the increase of the acyl chain length. The activity of r-LipA against *p*NP acetate (C_2_) was 4.29 ± 0.03 U/mg, but decreased to 1.38 ± 0.04 U/mg on *p*NP decanoate (C_10_) and 0.73 ± 0.01 U/mg on *p*NP dodecanoate (C_12_). The protein had almost no activity against *p*NP myristate (C_14_), *p*NP palmitate (C_16_) or *p*NP stearate (C_18_). To detect the kinetic parameters on various *p*NP esters with acyl chains of different lengths from 2 to 12 carbon atoms were examined (Table 5). The *k*_cat_ values consistently decreased and the *K*_m_ values increased with the increases in the acyl chain length of the substrates. Among the various *p*NP esters, the r-LipA had the lowest *K*_m_ value and the highest *k*_cat_/*K*_m_ value towards *p*NP acetate at 30 °C indicated that *p*NP acetate was the best substrate for r-LipA (*k*_cat_/*K*_m_, 186.643 s^−1^mM^−1^). At 4 °C, the *K*_m_ value of r-LipA towards *p*NP acetate was reduced to 0.037 ± 0.001 mM, which indicated that the affinity of the enzyme to *p*NP acetate increased at low temperature. Many lipases, such as the lipases from mesophilic *Geotrichum* sp. SYBC WU-3 [36] and *Candida albicans* [37], are capable of hydrolyzing water-soluble esters with short chain acyl group. The values for kinetic parameters reveal that short chain acyl group *p*NP ester (C_2_) is better substrate for r-LipA. Moreover, the catalytic constants of *k*_cat_/*K*_m_ towards short chain acyl group *p*NP esters (C_2_ and C_4_) are very high, so it is obvious that our r-LipA has high catalytic efficiency to hydrolyze esterase substrates.

It is interesting to see that the specific activity of r-LipA toward different *p*NP fatty acyl esters changed under different temperature and pH conditions (Table 4). At pH 8.0 and 30 °C, the activity of r-LipA on *p*NP butyrate (C_4_) was approximately 60% of that on *p*NP acetate (C_2_). When the reaction condition was changed to pH 9.0 and 30 °C, the activities of r-LipA on *p*NP butyrate (C_4_) and *p*NP acetate (C_2_) were nearly similar, *i.e.* approximately 86% and 83% of that on *p*NP acetate (C_2_) at pH 8.0 and 30 °C, respectively. For *p*NP butyrate (C_4_), the optimal activity was at pH 9.0 and 30 °C. At pH 9.0 and 40 °C, the maximum activity of r-LipA was on *p*NP octanoate (C_8_), which was approximately 74% of that on *p*NP acetate (C_2_) at pH 8.0 and 30 °C. Similarly, the optimal activities on *p*NP decanoate (C_10_) and *p*NP dodecanoate (C_12_) were at pH 9.0 and 30 °C or 40 °C, but the relative activities of both were still less than half of that on *p*NP acetate (C_2_) at pH 8.0 and 30 °C. These results indicated that higher pH values or temperatures shifted the optimal hydrolytic activity of the enzyme to the *p*NP substrates with longer chain fatty acyl esters. However, the promotion of temperature and pH value had small effects on those long chain *p*NP substrates, such as *p*NP myristate (C_14_), *p*NP palmitate (C_16_), and *p*NP stearate (C_18_) (Table 4). While the optimal substrate of the enzyme is *p*NP acetate (C_2_) under many different temperature and pH conditions, the best substrate changed to *p*NP octanoate (C_8_) under higher pH and temperature condition (pH 9.0, 40 °C). This few-researched characteristic paves the way for further researches of this cold-adapted lipase. Therefore, catalytic behaviors of cold-adapted enzymes at low temperatures are very interesting and worthy of future in depth experiments.

## 3. Experimental Section

### 3.1. Strains, Plasmids and Chemicals

The *Sorangium cellulosum* So0157-2 strain (the deposition ID in the China Center of Typical Culture Collection (CCTCC) is CCTCC M 208078) was cultured in liquid M26 medium at 30 °C as previously described [38]. *E. coli* Top10 and BL21 (DE3) were used as the cloning host and the expression host, and pMD-T simple vector (Takara, Dalian, China) and pET-22b (+) (Novagen, Darmstadt, Germany) were used as the cloning vector and the expression vector, respectively. *E. coli* cells harboring the recombinant plasmid were grown in Luria–Bertani (LB) broth supplemented with 100 μg/mL ampicillin [39]. The culture temperature was 30 °C for *S. cellulosum* strains and at 37 °C for *E. coli* strains.

The restriction enzymes, DNA polymerase, T4 DNA ligase, molecular weight protein marker and calf intestinal alkaline phosphatase used in this study were purchased from Fermentas (Burlington, Canada). The Plasmid Miniprep Kit, PCR purification kit and Genome extract kit used were purchased from Omega Bio-Tek (Norcross, USA) and used according to the manufacturer’s instructions. The substrates *p*-nitrophenyl (*p*NP) acetate (C_2_), butyrate (C_4_), caprylate (C_8_), decanoate (C_10_), laurate (C_12_), myristate (C_14_), palmitate (C_16_), stearate (C_18_), and 1,2-di-*O*-lauryl-rac-glycero-3-glutaric acid 6′-methylresorufin ester (DGGR) were purchased from Sigma (Aldrich, St. Louis, MO, USA). All other chemicals used were analytical grade unless otherwise specified.

### 3.2. Cloning and Sequence Analysis

The primer pair of LipA-F 5′-GGAATTCCATATGATGCCCGCGGACACCTTCACGTTTCAG-3′ and LipA-R 5′-CCCAAGCTTGCCGGCCGCGGCGCCGGCGC-3′ was designed on the base of the sequence information of *S. cellulosum* So ce56 (GenBank: AM746676). The restriction enzyme sites of *Nde* I and *Hind* III (underlined) were integrated to generate a His-tag. The reaction mixture was subjected to initial denaturation at 95 °C for 3 min, followed by 30 cycles of denaturation at 95 °C for 30 s, annealing at 63 °C for 30 s, and extension at 72 °C for 1.5 min and final extension at 72 °C for 10 min. The PCR products with the appropriate size were gel purified, ligated into the pMD-T simple vector. The *E. coli* Top10 cells harboring the recombinant plasmid pMD-*lipA* were then selected using LB agar plates supplemented with 5-bromo-4-chloro-3-indolyl-β-d-galactopyranoside (X-gal), isopropylthio-β-d-galactopyranoside (IPTG), and ampicillin. The cloned gene was confirmed by sequencing.

Sequence analysis and database similarity searches were on-line performed at the National Center for Biotechnology Information (NCBI) [40]. Multiple sequence alignments were performed using the ClustalW program [25], and the signal peptide sequence was deduced by SignalP 3.0 [19].

### 3.3. Construction of Recombinant Plasmid

The construction of recombinant plasmid was performed according to the method described by Sambrook and Russell [39]. Briefly, the confirmed recombinant plasmid pMD-*lipA* was digested by *Nde*I and *Hind*III restriction endonucleases and the product was purified and inserted into the plasmid pET-22b (+), which had been digested with the same enzymes. The recombinant plasmid, named pET-*lipA* was cloned and confirmed by sequencing.

### 3.4. Expression and Purification of the r-LipA

The recombinant product pET-*lipA* was primarily transformed into the cloning host *E. coli* Top10 and further into the expression host *E. coli* BL21 (DE3). *E. coli* BL21 (DE3) carrying pET-*lipA* was shaken overnight at 37 °C in LB broth supplemented with 100 μg/mL ampicillin. The culture was then inoculated (1%) into fresh LB broth containing 100 μg/mL ampicillin and grown at 37 °C until the absorption at 600 nm reached 0.6. Then, IPTG was added at a final concentration of 0.5 mM and the culture was incubated for additional 4 h at 37 °C for the induction of the expression of r-LipA. The cells were harvested by centrifugation at 8000× *g* for 5 min, washed twice in ice-cold lysis buffer (50 mM Tris-HCl, 250 mM NaCl, pH 8.0) and resuspended in the same buffer. After ultrasonic disruption, lysates were centrifuged (12,000 rpm at 4 °C for 30 min) to remove cell debris. The supernatant was passed through a 0.45-μm filter and then applied to metal-chelating Ni-NTA affinity chromatography (GE Healthcare) that had been equilibrated with lysis buffer. After washed in washing buffer (50 mM Tris-HCl, 250 mM NaCl, 50 mM imidazole, pH 8.0), the Ni-NTA bound r-LipA was eluted using elution buffer (50 mM Tris-HCl, 250 mM NaCl, 250 mM imidazolea, pH 8.0) and dialyzed against a 50-mM Tris-HCl buffer (pH 8.0), obtaining purified r-LipA proteins. Polyacrylamide gel electrophoresis in the presence of sodium dodecyl sulfate (SDS-PAGE) was performed to check the expression and purification according to the method described by Sambrook and Russell [39]. *E. coli* cells containing pET-22b (+) induced by IPTG was used as negative control.

After SDS-PAGE, the purified protein band was excised, digested with trypsin and analyzed by liquid chromatography-electrospray ionization-tandem mass spectrometry (LC-ESI-MS/MS). The resulting peptide sequences were compared to the r-LipA amino acid sequence.

### 3.5. Lipase Activity Assay

Lipase activity was determined by measuring the release of *p*NP using the chromometer method with some modifications [11]. The reaction mixture contained 0.5 mM *p*NP ester in ethanol, 50 mM Tris-HCl buffer (pH 8.0) containing 1% ethanol, and 10 μL of enzyme in a final volume of 1 mL. The mixture was incubated at 30 °C for 10 min. Absorbance was then measured at 410 nm. One unit of enzyme activity was defined as the release of 1 μmol of *p*-nitrophenol per min. Protein concentration was determined by Bradford’s method using bovine serum albumin as standard. All measurements were carried out in triplicate. The values were the mean of the data.

To determine lipase activity of the protein, the triglyceride derivative DGGR was used as a chromogenic substrate, according to the previously described methods [6,24,41]. The compound is cleaved by lipase, resulting in an unstable dicarbonic acid ester, which is spontaneously hydrolyzed to yield glutaric acid and methylresorufin, a bluish-purple chromophore with peak absorption at 580 nm. *Candida rugosa* lipase (Sigma, Aldrich, St. Louis, MO, USA) was used as positive control. The background hydrolysis of the substrate was deducted by using a reference sample of identical composition to the incubation mixture omitted lipase. It was shown that the specificity of DGGR for pancreatic lipase compares well with that of the widely established turbidimetric method [6,24]. Lipase activity was expressed as U/L (μmoles of substrate converted per minute per liter under the specified conditions).

### 3.6. Effects of Temperature on the r-LipA Avtivity and Stability

The optimum temperature was determined by assaying the lipase activity of r-LipA in a 50 mM Tris-HCl buffer (pH 8.0) under the standard assay conditions in a temperature range from 0 °C to 60 °C. The thermostability of r-LipA was determined by measuring the residual activity under standard assay conditions following pre-incubating the enzyme solution at 40 °C, 50 °C, 55 °C and 60 °C for various periods of time in the absence of substrate. The activity of r-LipA without pre-incubation was set as 100%.

### 3.7. Effects of pH on the r-LipA Avtivity and Stability

For determining the optimum pH of r-LipA, the pH of the reaction mixtures was varied from 3.0 to 10.5 using different pH buffers (McIlvaine buffer for pH values of 3.0–7.5; 50 mM Tris-HCl for pH values of 7.5–10.5). Similarly, to determine pH stability, r-LipA was incubated in buffers of various pH values (pH 3.0–10.5) for 8 h at 4 °C before the measurement of the residual lipase activities under standard assay conditions.

### 3.8. Effect of Metal Ions, Detergents and Organic Solvents on the r-LipA Avtivity

The effects of different metal ions, detergents on enzymatic activity were assessed in 50 mM Tris-HCl buffer (pH 8.0) at 30 °C. The reactions contained various metal ions (LiCl, NaCl, KCl, MgCl_2_, CaCl_2_, CrCl_3_, MnCl_2_, CoCl_2_, ZnCl_2_, HgCl_2_, FeSO_4_, CuSO_4_, NiSO_4_) and ethylenediamine tetraacetic acid (EDTA) at a final concentration of 1 or 10 mM; 0.1% or 1% (w/v) sodium dodecyl sulfate (SDS), cetyltrimethyl ammonium bromide (CTAB), Tween 20, Tween 80 or Triton X-100 and then the lipase activity was measured under standard conditions (30 °C, 10 min). The activity of r-LipA determined in the buffer with no addition of the metal ions or detergents was set as 100%.

Effect of various organic solvents on the lipase activity was investigated at the concentration of 25% (v/v, *i.e.*, mixing 1 mL of organic solvent in 3 mL of the enzyme solution). The reaction mixture was incubated at 30 °C with shaking at 150 rpm for 2 h. Tolerance of the enzyme against solvent was assayed under standard conditions, and the remaining activity was expressed as the ratio to the lipase activity with distilled water instead of solvent.

### 3.9. Substrate Specificity of the r-LipA

The substrate specificity of the purified r-LipA protein was performed using the following substrates of *p*NP esters: acetate (C_2_), butyrate (C_4_), caprylate (C_8_), decanoate (C_10_), laurate (C_12_), myristate (C_14_), palmitate (C_16_), stearate (C_18_). Enzyme assays were performed in 50 mM Tris-HCl (pH 8.0) under standard conditions (30 °C, 10 min). Furthermore, the enzymatic activities toward different *p*NP esters were performed under different temperature and pH conditions. The reaction pH was in McIlvaine buffer (0.2 M Na_2_HPO_4_/0.1 M citric acid, pH 7.0) or 50 mM Tris-HCl buffer (pH 8.0 and 9.0), the temperature was at 20 °C, 30 °C, or 40 °C. The highest activities of enzyme assay using the substrate (*i.e.*, *p*NP acetate) at 30 °C was defined as the 100%.

The *K*_m_, *V*_max_, and *k*_cat_ values of the purified r-LipA were determined by measuring the enzyme activity in 50 mM Tris-HCl (pH 8.0) at 4 °C (with 0.025–4 mM *p*NP acetate as the substrate) and 30 °C (with 0.025–4 mM *p*NP acetate, *p*NP butyrate, *p*NP caprylate, *p*NP decanoate or *p*NP laurate as the substrate), respectively. All experiments were performed in triplicate. The *K*_m_ and *V*_max_ values for the r-LipA were estimated from Lineweaver-Burk plots using the non-linear regression computer program GraFit 7.0 software.

## 4. Conclusions

In this study, we report the cloning of a novel lipase gene, *lipA*, from *S. cellulosum* So0157-2. Biochemical characterization showed that the r-LipA was a novel cold-adapted lipase and possessed several attractive characteristics. The lipase showed high activity in broad ranges of pH and temperature, and also, stability towards many additives including metal ions, detergents, and organic solvents. As a typical cold-adapted lipase, r-LipA is most active at low and intermediate temperatures, which may offer some advantages over the currently used lipases in many processes requiring low to moderate temperature. These characteristics are of interest for application in the detergent industry or organic synthesis. The results also demonstrate that *S. cellulosum* is a highly promising source for screening of lipases for biotechnological applications.

## Figures and Tables

**Table 1:** Effects of various metal ions on the activity of r-LipA. The reactions contained various metal ions at a final concentration of 1 or 10 mM and then the lipase activity was measured under standard conditions (30 °C, 10 min). The activity of r-LipA determined in the buffer with no addition of the metal ions was set as 100%. Values are means ± SD from three independent experiments.

Metal ions	Relative remaining activity (%) of r-LipA	
	1 mM	10 mM
Control	100.0 ± 3.0	100.0 ± 1.6
Li^+^	96.5 ± 1.2	93.3 ± 1.5
Na^+^	97.1 ± 3.8	105.9 ± 5.5
K^+^	94.3 ± 0.6	96.0 ± 4.7
Mg^2+^	105.1 ± 3.8	126.1 ± 2.5
Ca^2+^	112.6 ± 1.5	140.2 ± 0.3
Fe^2+^	140.8 ± 2.4	77.1 ± 2.4
Mn^2+^	120.1 ± 1.9	103.4 ± 0.9
Cr^3+^	110.2 ± 3.0	59.5 ± 1.9
Co^2+^	104.1 ± 1.8	99.2 ± 1.0
Ni^2+^	97.6 ± 1.9	63.3 ± 2.6
Cu^2+^	57.8 ± 1.5	4.0 ± 0.8
Zn^2+^	56.8 ± 0.9	42.9 ± 4.8
Hg^2+^	88.4 ± 2.2	40.1 ± 1.5
EDTA	109.8 ± 1.5	99.8 ± 1.4

**Table 2:** Effects of various detergents on the activity of r-LipA. The reactions contained 0.1% or 1% (w/v) sodium dodecyl sulfate (SDS), cetyltrimethyl ammonium bromide (CTAB), Tween 20, Tween 80 or Triton X-100 and then the lipase activity was measured under standard conditions (30 °C, 10 min). The activity of r-LipA determined in the buffer with no addition of the detergents was set as 100%. Values are means ± SD from three independent experiments.

Detergents	Relative remaining activity (%) of r-LipA	
	0.1%	1%
Control	100.0 ± 2.9	100.0 ± 2.6
SDS	10.2 ± 0.8	9.0 ± 0.7
CTAB	203.7 ± 3.1	95.4 ± 1.4
Tween 20	139.2 ± 0.8	113.0 ± 5.5
Tween 80	132.4 ± 2.2	119.4 ± 2.2
Triton X-100	146.8 ± 0.4	196.3 ± 2.1

**Table 3:** Effects of various organic solvents on the activity of r-LipA. The reaction mixture contained 25% (v/v, *i.e.*, mixing 1 mL of organic solvent in 3 mL of the enzyme solution) organic solvents was incubated at 30 °C with shaking at 150 rpm for 2 h. After that, tolerance of the enzyme against solvent was assayed under standard conditions, and the remaining activity was expressed as the ratio to the lipase activity with distilled water instead of solvent. Values are means ± SD from three independent experiments.

Organic solvents	log *P*	Relative remaining activity (%) of r-LipA	
		30 min incubation	2 h incubation
Control	-	100.0 ± 1.6	100.0 ± 2.1
Dimethyl sulfoxide	−1.22	97.1 ± 0.3	88.1 ± 2.2
Methanol	−0.76	97.7 ± 2.5	90.2 ± 1.4
Ethanol	−0.24	75.4 ± 3.1	67.6 ± 1.1
Acetone	−0.23	78.8 ± 1.3	69.2 ± 2.7
Isopropanol	0.14	57.2 ± 3.1	46.6 ± 2.8
*n*-Propanol	0.28	76.3 ± 1.7	50.2 ± 0.7
*n*-Butanol	0.8	99.2 ± 4.5	82.6 ± 1.6
Diethylether	0.85	113.7 ± 2.0	118.5 ± 1.8
Chloroform	2	139.0 ± 1.1	142.8 ± 3.5
Benzene	2	140.5 ± 5.5	160.2 ± 3.3
Toluene	2.5	150.9 ± 0.6	159.2 ± 0.9
*p*-Xylene	3.1	153.7 ± 5.2	152.9 ± 2.5
Cyclohexane	3.2	156.0 ± 3.7	136.9 ± 0.8
*n*-Hexane	3.5	153.0±2.8	130.8 ± 3.5
*n*-Heptane	4	159.1 ± 3.4	167.1 ± 1.2
Isooctane	4.7	163.7 ± 0.5	132.8 ± 0.7

**Table 4:** Catalytic activities of r-LipA on different substrates under different pH values and temperatures. The reaction pH was in McIlvaine buffer (0.2 M Na_2_HPO_4_/0.1 M citric acid, pH 7.0) or 50 mM Tris-HCl buffer (pH 8.0 and 9.0), the temperature was at 20 °C, 30 °C, or 40 °C. The highest activities of enzyme assay using the substrate (*i.e.*, *p*NP acetate) at 30 °C was defined as the 100%. Values are means ± SD from three independent experiments.

Substrates	Relative remaining activity (%)								
	pH 7.0, 20 °C	pH 7.0, 30 °C	pH 7.0, 40 °C	pH 8.0, 20 °C	pH 8.0, 30 °C	pH 8.0, 40 °C	pH 9.0, 20 °C	pH 9.0, 30 °C	pH 9.0, 40 °C
*p*NP acetate	16.6 ± 0.3	32.6 ± 1.5	43.2 ± 4.5	71.6 ± 2.2	100.0 ± 2.6	60.5 ± 1.6	72.5 ± 1.4	86.1 ± 1.6	60.8 ± 0.8
*p*NP butyrate	7.9 ± 3.0	18.3 ± 2.3	26.2 ± 3.9	38.4 ± 1.3	63.4 ± 2.3	53.3 ± 2.1	54.1 ± 3.6	83.3 ± 1.0	64.6 ± 1.4
*p*NP octanoate	8.1 ± 1.2	12.8 ± 1.8	15.7 ± 2.1	33.3 ± 0.5	52.5 ± 0.3	52.1 ± 2.9	41.5 ± 2.5	68.4 ± 2.3	74.2 ± 2.6
*p*NP decanoate	1.4 ± 0.2	4.9 ± 3.0	5.9 ± 0.5	22.2 ± 0.7	32.1 ± 0.9	24.7 ± 3.0	24.0 ± 0.2	40.4 ± 2.0	44.7 ± 2.8
*p*NP dodecanoate	1.6 ± 0.3	2.5 ± 0.1	5.8 ± 1.4	13.2 ± 5.2	17.0 ± 1.0	20.5 ± 1.8	23.3 ± 1.8	43.9 ± 1.5	40.0 ± 1.0
*p*NP myristate	1.6 ± 1.0	2.2 ± 1.4	5.7 ± 2.9	5.7 ± 1.3	3.9 ± 2.4	5.6 ± 2.2	5.4 ± 2.7	5.2 ± 3.0	4.8 ± 3.4
*p*NP palmitate	1.6 ± 0.3	1.9 ± 1.6	4.1 ± 0.6	2.0 ± 1.2	2.3 ± 0.8	1.3 ± 0.8	2.6 ± 2.3	5.2 ± 0.1	1.3 ± 1.3
*p*NP stearate	1.3 ± 1.0	1.0 ± 0.1	2.5 ± 0.6	3.4 ± 0.1	1.9 ± 1.5	0.7 ± 0.8	2.5 ± 0.1	5.3 ± 2.5	1.9 ± 0.4

**Table 5:** Kinetic parameters for r-LipA on p-nitrophenyl esters.

Substrates	Temperature (°C)	*K*_m_ (mM)	*k*_cat_ (s^−1^)	*k*_cat_/*K*_m_ (s^−1^·mM^−1^)
*p*NP acetate (C_2_)	4	0.037 ± 0.001	7.008 ± 0.100	186.643 ± 3.201
*p*NP acetate (C_2_)	30	0.174 ± 0.006	29.225 ± 0.977	168.114 ± 2.097
*p*NP butyrate (C_4_)	30	0.366 ± 0.211	13.617 ± 0.735	37.173 ± 0.128
*p*NP octanoate (C_8_)	30	0.704 ± 0.026	3.294 ± 0.114	4.677 ± 0.013
*p*NP decanoate (C_10_)	30	1.664 ± 0.047	1.565 ± 0.036	0.940 ± 0.005
*p*NP dodecanoate (C_12_)	30	1.946 ± 0.059	1.205 ± 0.032	0.619 ± 0.002
